# Development of a Bayesian response-adaptive trial design for the Dexamethasone for Excessive Menstruation study

**DOI:** 10.1177/0962280215606155

**Published:** 2015-09-30

**Authors:** Christian Holm Hansen, Pamela Warner, Richard A Parker, Brian R Walker, Hilary OD Critchley, Christopher J Weir

**Affiliations:** 1 MRC Tropical Epidemiology Group, London School of Hygiene & Tropical Medicine, London, UK; 2 Centre for Population Health Sciences, University of Edinburgh, Edinburgh, UK; 3 Edinburgh Clinical Trials Unit, University of Edinburgh, Edinburgh, UK; 4 Edinburgh Health Services Research Unit, Edinburgh, UK; 5 British Heart Foundation Centre for Cardiovascular Science, University of Edinburgh, Edinburgh, UK; 6 MRC Centre for Reproductive Health, University of Edinburgh, Edinburgh, UK

**Keywords:** Dose-finding, normal dynamic linear model, adaptive design, trial design development, simulation

## Abstract

It is often unclear what specific adaptive trial design features lead to an efficient design which is also feasible to implement. This article describes the preparatory simulation study for a Bayesian response-adaptive dose-finding trial design. Dexamethasone for Excessive Menstruation aims to assess the efficacy of Dexamethasone in reducing excessive menstrual bleeding and to determine the best dose for further study. To maximise learning about the dose response, patients receive placebo or an active dose with randomisation probabilities adapting based on evidence from patients already recruited. The dose-response relationship is estimated using a flexible Bayesian Normal Dynamic Linear Model. Several competing design options were considered including: number of doses, proportion assigned to placebo, adaptation criterion, and number and timing of adaptations. We performed a fractional factorial study using SAS software to simulate virtual trial data for candidate adaptive designs under a variety of scenarios and to invoke WinBUGS for Bayesian model estimation. We analysed the simulated trial results using Normal linear models to estimate the effects of each design feature on empirical type I error and statistical power. Our readily-implemented approach using widely available statistical software identified a final design which performed robustly across a range of potential trial scenarios.

## 1 Introduction

Adaptive designs generally offer added flexibility and efficiency over conventional trial designs. However, it is difficult to select the design options that provide an effective adaptive design which is also feasible to implement. Before deciding on a particular design, it is generally advisable to carry out a simulation study.

The methodology of Bayesian adaptive designs has grown steadily in recent years; alongside this, publications providing guidance on simulation studies for the development of Bayesian response-adaptive designs^1^ are a valuable resource. Characterising the properties of competing adaptive designs and choosing a ‘best design’ to take forward is not a trivial task. In this paper, we describe our experiences from a simulation study which was conducted to develop the response-adaptive dose-finding study design for the MRC-funded Dexamethasone for Excessive Menstruation trial (DexFEM; MRC reference: MR/J003611/1).

## 2 Outline

A brief account of the background and motivation for the DexFEM study is provided in section 3 along with a description of the methods planned for the analysis of this adaptive trial. The design development study is introduced in section 4 which gives an account of the design options and scenarios that are investigated in the trial simulations. Sections 5 and 6 give details of the simulation engine and the methods used to summarise the simulated trial results. The findings from the design development study are presented in section 7. Section 8 explains the subsequent choice of the adaptive design for DexFEM followed by more general discussion of our approach in a wider context in section 9.

## 3 The DexFEM study

Heavy Menstrual Bleeding (HMB) is a common problem, yet existing medical treatments are often ineffective. Surgical treatments such as hysterectomy, are unacceptable to many, particularly those who wish to preserve their fertility. DexFEM aims to establish if Dexamethasone (a synthetic glucocorticoid) taken orally is effective in reducing menstrual blood loss (MBL) in women with heavy menstrual bleeding.

Women with HMB may have glucocorticoid deficiency in the lining of the womb (endometrium) and as a consequence impaired blood vessel function and increased menstrual blood loss.^2^ We propose “rescue” of such a deficiency could reduce excessive menstrual bleeding. The published DexFEM protocol provides further details of the trial.^3^

The optimal dose for achieving efficacy and few adverse effects is unknown but is likely to be between 0.4 mg and 1.8 mg per day. The aims of the DexFEM trial are: (1) to establish whether Dexamethasone in this dose range is efficacious in reducing menstrual blood loss and (2) to identify which is the best dose for further study in a later trial.

To estimate the dose-response curve we aim to enrol up to 108 women with HMB to achieve 100 completers in a response-adaptive, multi-arm, parallel-group dose-finding trial. Participants are randomised to receive either placebo or one of several active doses in the range 0.4 mg to 1.8 mg. Allocation probabilities are equal across the active doses at the start of recruitment but will be updated for subsequent trial participants as outcome data begin to accrue and information is gathered about the likely shape of the dose-response curve. This strategy has the advantage that more information is obtained in the critical region of the underlying dose-response curve and fewer women are randomised to less effective doses. To limit potential bias in the randomised comparisons at the end of the study due to a drift in participant characteristics across time, the placebo allocation rate will remain constant throughout the study. Thus, the allocation rate to placebo is the only one that is not subject to adaptation.

Two characteristics of menstrual bleeding research are relevant to DexFEM. (1) *Cycle.* The symptoms of HMB occur only cyclically according to the pattern of menstruation (most usually 5 to 8 days menstruation starting every 26 to 29 days, but variable even within women). In DexFEM, we wish to trial three cycles of treatment. (2) *Outcome*. Following collection of sanitary protection by the patient, laboratory estimation of menstrual blood loss volume for the period can be achieved within a few days of the end of the period making it a suitable outcome for use in an adaptive design. In DexFEM only the second and third treated cycles involve menstrual blood volume measurement.

### 3.1 Modelling the dose-response

The dose-response curve is estimated using Bayesian methods in a second order Normal Dynamic Linear Model^4^ (NDLM). Such models offer flexibility over non-dynamic models as few restrictions are placed on the shape of the estimated curve. NDLMs also lead to efficiency gains since the treatment effect estimate at a given dose is also informed by that of neighbouring doses.

The NDLM is specified in terms of an observation equation and an evolution equation. Our observation equation

$$Y_{i}=\theta_{j}+\beta {\mathrm{Ybl}}_{i}+\nu_{i}i=1,\ldots ,100;j=1,\ldots ,J;\nu_{i}\;∼i.i.d.N(0,\sigma_{\nu }^{2})$$

models *Y_i_*, the MBL change from baseline to follow-up in subject *i*, as a linear function of the mean-centred baseline MBL *Ybl_i_*, and *θ_j_*, the treatment effect at dose level *j*. *β* is the regression coefficient for *Ybl_i_* and *ν_i_* is the observational error term.

**Figure 1.**
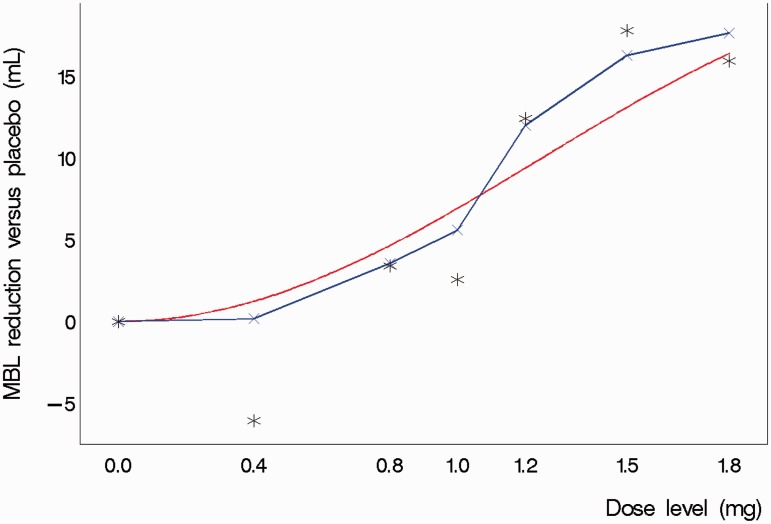
Illustration of a Normal Dynamic Linear Model fit to simulated trial data based on a theoretical dose–response relationship. The figure shows the theoretical dose-response curve, the piecewise linear fit derived from the Normal Dynamic Linear Model and the actual observed means from a single simulated trial realisation (stars).The outlying mean at 0.4 mg was based on just six patients randomised to this dose and consequently had a relatively smaller influence on the model estimate of the treatment effect at that dose.

The evolution equation

$$\theta_{j}=\theta_{j-1}+\delta_{j-1}+\omega_{j}\delta_{j}=\delta_{j-1}+{ɛ}_{j}j=1,\ldots ,J;\omega_{j},{ɛ}_{j}∼i.i.d.N(0,\sigma_{\omega }^{2})$$

equates the treatment effect at dose level *j* to the treatment effect at the previous dose level plus a systematic deviation, *δ_j__ __−__ _*_1_, and an evolution error, *ω_j_*. Finally *δ_j_*, the change in treatment effect from dose level *j* to dose level *j + *1*,* is essentially modelled as a random walk with step size governed by *N(δ_j__ __−__ _*_1_*, σ_ω_*^2^*)*. The model formulation therefore assumes linear changes in the treatment effect from one dose level to the next but avoids restricting the overall curve to follow a particular parametric specification or even monotonicity (Figure 1). *ν_i_*, *ω_j_* and *ɛ_j_* are mutually as well as internally independent. *ω_j_* and *ɛ_j_* are restricted to have the same variance to limit the number of parameters needing estimated with this relatively small sample size. Similarly, the observation and evolution error variances are assumed constant for all subjects and dose levels.

We used the freely available WinBUGS software^5^ to estimate this system of equations using Bayesian methods. We defined the variance of the evolution error as a multiple of the observational error variance such that *σ_ω_*^2^*^ ^= Wσ_ν_*^2^. A uniform *U*(0.001, 100) prior distribution was specified for *W* and a vague half-normal prior distribution *N*(0, 100), defined only on the non-negative range of the scale, was placed on *σ_ν_*^2^. We also specified prior *N*(0, 10000) distributions for *θ*_0_, *δ*_0_, and *β*.

### 3.2 Adaptation after interim analyses

A conventional design might randomise all trial participants evenly across the active dose range before deriving, at the end, a final model estimate of the dose-response curve. With our adaptive design we will, while recruitment is still ongoing, apply the NDLM to the available outcome data already accrued to obtain intermediate estimates of the dose-response curve. Evidence of emerging trends can then inform adaptation of randomisation probabilities to the active doses for future trial participants in a way that optimises estimation of the dose-response curve according to some pre-defined criterion. Typically as the trial progresses, more participants are randomised to doses which are emerging as more effective so that the dose-response curve in the range containing these doses may be estimated with greater precision than would have been the case in a conventional design.

The criterion on which adaptation is based might be a simple rule, for example to allocate future participants using probabilities that are proportional to current effect estimates at each dose level. It might also be a more complex function of the predicted utility of future randomisations at each dose level, for example using a measure of the precision with which the optimal dose level is estimated, or alternatively, a measure of the precision with which efficacy is estimated at the optimal dose level. The criterion should reflect the trial objectives and is likely to differ depending on whether the primary interest is to obtain a robust estimate of the dose-response curve across the entire dose range or just around the optimum dose level. As our second aim is to identify the best dose for further study, we applied a commonly used definition of the optimal dose: the ED95, the smallest dose which achieves at least 95% of the maximum treatment effect.

## 4 The design development study

We aimed to develop a feasible adaptive design: that is, one with a limited number of scheduled adaptations and a specified number of possible doses (to allow the pharmacy to prepare capsules in advance). A balance was therefore needed between satisfying these feasibility criteria and obtaining effective adaptation. To this end, we considered several different designs and assessed the suitability of each for DexFEM in a comprehensive pre-trial simulation study. The aim of the design development study was to generate simulated trial results to characterise the properties of candidate designs in terms of type I error and statistical power and ultimately to identify one design which would perform well across a broad range of scenarios.

### 4.1 Design options

#### 4.1.1 Dose levels

A daily Dexamethasone dose of 1.5 mg was used in two small pilot trials, the dose being selected based on experience with the drug in other indications and its planned use for 5-day treatment across repeated menstrual cycles. No safety concerns emerged. The adaptive trial provides an opportunity to estimate the dose-response curve over as wide a dose range as possible. Clinical judgment was that 1.8 mg was the maximum dose that should be tested, so the daily dose levels selected for study were 0.4 mg, 0.8 mg, 1.0 mg, 1.2 mg, 1.5 mg and 1.8 mg.

It was not clear how many doses we could reliably study in the trial. Would a design with the maximum practical number of doses perform better since intervals between observed points on the dose-response curve are shorter, or would a design with fewer doses perform better since better precision would be achieved in the treatment effect estimates at each dose level? We investigated this in the design development study (Table 1).

**Table 1.** Design options and trial scenarios investigated in the design development study.

1. Design options
1.1 Active dose levels to include for investigation
(a) 0.4 mg, 0.8 mg, 1.0 mg, 1.2 mg, 1.5 mg, 1.8mg
(b) 0.4 mg, 1.0 mg, 1.5 mg, 1.8mg
1.2 Timing of adaptations (in terms of number of patients randomised)
(a) 33
(b) 50
(c) 66
(d) 10, 35 and 60
(e) 20, 45 and 70
(f) 49, 66 and 83
(g) 12, 24, 36, 48 and 60
(h) 16, 32, 50, 66 and 84
(i) 44, 55, 66, 77 and 88
(j) (no adaptation)
1.3 Placebo allocation rate *(in design with J-*1 *active doses)*
(a) 1/J
(b) 2/J
1.4 Criterion for adaptation *(allocation probabilities are)*
(a) scaled in proportion to probability that dose is efficacious (play-the-winner)
(b) based on predicted information gain resulting from a future randomisation at each dose

2. Scenarios
2.1 Variance of intra-participant MBL measurements
(a) σ_e_^2 ^= (17.9 mL)^2^
(b) σ_e_^2 ^= (22.0 mL)^2^
(c) σ_e_^2 ^= (26.0 mL)^2^ (only used when simulating heteroscedasticity)
2.2 Shape of dose-response curve
(a) Steep incline towards higher end of dose-range
(b) Slow incline with increasing dose levels
(c) Non-monotonic with maximum at 1.2 mg
(d) Flat (i.e. no effect)
2.3 Magnitude of effect at maximum effective dose
(a) 8.2 mL
(b) 16.4 mL
2.4 Heteroscedasticity
(a) Absent
(b) Present
2.5 Mechanism of treatment
(a) Effect independent of baseline MBL
(b) Effect magnitude proportionate to baseline MBL
2.6 Enrolment rate
(a) 2 participants per month
(b) 4 participants per month

#### 4.1.2 Number and spacing of adaptations

In principle, the ideal design would encompass model re-estimation and subsequent adaptation of future allocation probabilities to each of the active doses with every new MBL measurement collected. In practice, a fully sequential design of this sort would be unnecessarily challenging and work-intensive and with little efficiency gain over a simpler design with fewer adaptations (based on what we learned from initial exploratory simulation work). We assessed the performance of designs with one, three and five adaptations over the course of recruitment as well as non-adaptive designs (zero adaptations) for comparison. Within each design, adaptations were scheduled at pre-defined points during the recruitment phase. An example of a design with one adaptation [see Table 1, section 1.2(b)] is a trial where the first 50 participants are allocated with equal probability to any of the dose levels (including placebo). An interim analysis is then carried out after the 50th participant has been randomised, following which allocation probabilities for subsequent participants are adapted to increase the potential for a successful trial outcome in light of the evidence collected so far. In this example the adaptation was scheduled exactly half-way through the 100 planned randomisations but would adaptations scheduled earlier or later in the recruitment phase perform better, or should they instead be spaced evenly between randomisations throughout recruitment? We assessed the performance of ten adaptation schedules [Table 1, section 1.2 (a)–(j)].

#### 4.1.3 Placebo allocation rate

The placebo allocation probability remained fixed throughout the trial. For a design with *J-*1 active doses we had initially planned to allocate participants to receive placebo with probability 1*/J*, such that participants during the initial phase of recruitment, i.e. before the first adaptation, would have equal probability of being allocated to any of the *J* treatment arms. However, in order to cover the range of values successfully implemented in previous multi-arm multi-stage adaptive trials^6,7^ we assessed the performance of designs using placebo allocation probabilities of both 1*/J* and 2*/J*.

#### 4.1.4 Criterion for adaptation of active dose randomisation probabilities

We evaluated two options for the choice of criterion for adaptation. One was the ‘play-the-winner’ rule where subsequent trial participants are randomly allocated to one of the active doses in proportion to the posterior probability that the treatment dose effects at least some reduction in MBL as evaluated in the interim analysis.

The second adaptation criterion considered was a utility function which quantifies the information gain from future randomisations at each dose level. The function we used was the predicted variance of the response at the current ED95 estimate after one future randomisation at each of the active doses. Subsequent trial participants were then randomised in proportion to the predicted increase in precision that would result from a randomisation at each dose.

To avoid computationally intensive nested MCMC routines, we used importance sampling^8^ as applied by Weir et al.^9^ to predict the variance of the response at ED95 after one future randomisation. At each dose, the predicted variance is based on M = 100 predictions for the future observed MBL difference and for each of these we estimate the posterior predictive distribution for the response at ED95 based on t = 10,000 iterations of the MCMC sampler.

#### 4.1.5 Other considerations

The response variable, *Y = Yfu − Ybl*, was the difference between the baseline MBL measurements obtained at screening, *Ybl*, and the follow-up MBL measurements, *Yfu,* collected during the treatment phases of the trial. Early simulation results suggested that we were able to reduce the variance of the modelled outcome, *Var(Y|Ybl)* by about 50% by collecting MBL measurements over two menstrual cycles at screening, *Y*_1_ and *Y*_2_, and similarly over two cycles at follow-up, *Y*_3_ and *Y*_4_, and letting *Ybl = (Y*_1_ + *Y*_2_*)/*2 and *Yfu = (Y*_3_ + *Y*_4_*)/*2. As these twin measurements at baseline and follow-up had been planned from the outset, and in light of the resultant reduction in variance, we decided not to reduce the number of menstrual cycles monitored during a woman's trial participation.

### 4.2 Scenarios

The above design options were assessed under a number of alternative assumptions for the true dose-response curve and other unknown parameters to ensure that the final design chosen would perform well under a broad range of scenarios. The choice of scenarios to be assessed was informed both by empirical data from previous studies and through careful elicitation of expert opinion from the clinical team guided by the Sheffield Elicitation Framework (SHELF^10^).

The variance of repeated intra-participant MBL measurements, *σ_e_*^2^, was likely to have a large effect on the probability of a successful trial. Data from an observational study^11^ suggested normally distributed within-participant errors with a variance of *σ_e_*^2^*^ ^= *17.9 mL. We also investigated scenarios with *σ_e_*^2^*^ ^= *22.0 mL and *σ_e_*^2^*^ ^= *26.0 mL. There was also some evidence from this source of increasing variance with greater MBL measurements. Design options were therefore investigated under both homo- and heteroscedastic errors. Heteroscedasticity was induced in the simulated within-participant errors through a simple parametric function of the baseline MBL (Appendix 1) based on empirical data.^11^

The likely mechanism of a treatment effect on participants' MBL was also considered. Treatment effects in medical research are often modelled as an absolute change independent of baseline levels of the outcome variable. In practice, the health improvement which results from a medical treatment is often dependent on the initial severity of the problem. Generally, treatment of a bigger problem results in greater improvements in absolute terms. Our investigation of candidate designs was therefore extended to include simulated scenarios where the treatment effect was 10% or 20% of the baseline MBL measurement at the most effective dose, for small and large simulated treatment effect magnitudes respectively.

We did not incorporate the modelling of heteroscedasticity and interactions between treatment effects and baseline MBL levels in the NDLM, yet these were included as plausible scenarios essentially to assess the robustness of the design for these scenarios where the model is misspecified.

**Figure 2.**
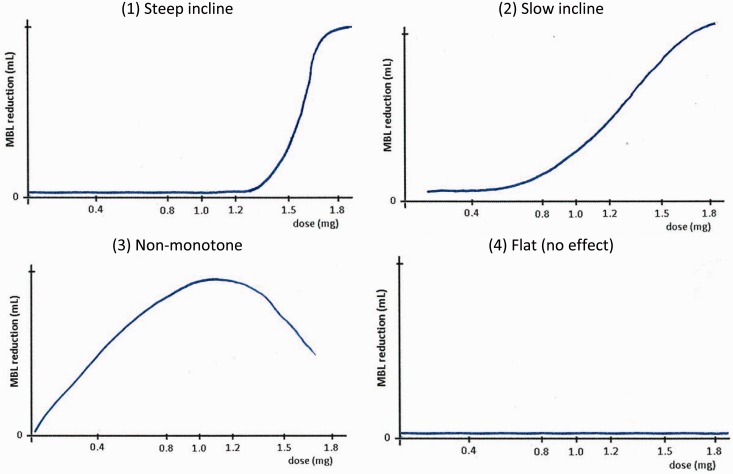
Four scenarios for the true dose-response relationship.

We also included a number of scenarios for the shape of the true dose-response curve and the magnitude of the treatment effect at the maximum-effective dose level (Figure 2). The trial enrolment rate was also believed to be of importance since participants randomised during the follow-up phase of other participants and during the time taken to carry out model re-estimation and adaptation, would not benefit from adaptation. Typically, a fast enrolment rate relative to the length of follow-up will reduce the benefits of using an adaptive design.

Table 1 shows the range of design options and scenarios investigated in the design development study. Four different design options were included as outlined above (1. dose levels, 2. timing and number of adaptations, 3. placebo allocation rate and 4. criterion for adaptation) and these were considered under various scenarios covering assumptions in six critical areas (1. variance of MBL measurements, 2. shape and 3. magnitude of the dose-response curve, 4. heteroscedastic error variance, 5. moderation of treatment effect by baseline MBL and 6. enrolment rate). With reference to Table 1, one example of a simulated trial would have the design options (1.1.a), (1.2.d), (1.3.c), (1.4.a) and scenario assumptions (2.1.b), (2.2.c), (2.3.b), (2.4.b), (2.5.b), (2.6.a), i.e. a trial with all six active dose levels included, three adaptations (one after each of 10, 35 and 60 randomisations), a 2/7 placebo allocation rate and a play-the-winner adaptation rule investigated under the scenario with MBL variance parameter of (22 mL)^2^, a non-monotonic dose-response curve with a treatment effect of 16.4 mL at the maximum effective dose, heteroscedastic error variance, treatment effect proportionate to baseline MBL and a trial enrolment rate of 2 participants per month.

## 5 Trial simulations

The performance of the many candidate designs was assessed under the various scenario options in a substantial simulation exercise using a fractional factorial setup. We simulated all aspects of a trial including screening, enrolment, randomisation, effects of treatment, repeated interim analyses (at pre-defined points during enrolment as specified by the design), adaptation of subsequent randomisation probabilities, and at the end of each trial, a final analysis. Each such trial simulation was repeated 200 times to ensure good precision in the performance characterisation of the design under the particular scenario. Independent data were generated for each simulated trial and design-scenario combination. Using a single control program we conducted the entire simulation exercise from within SAS software version 9.3 (SAS Institute Inc., Cary, NC, United States) which in turn invoked WinBUGS^5^ for Bayesian model estimation and empirical estimates of posterior distributions. (The paper by Zhang et al. (2008)^12^ is an excellent resource on how to control WinBUGS via a SAS program.)

At the core of the exercise was the simulation engine which generated the simulated trial data. We aimed to mimic a real-life trial as closely as possible by simulating screening data at the eligibility stage before enrolment. The DexFEM trial recruits participants who suffer from heavy menstrual bleeding. For the purpose of the trial this has been defined through the inclusion criteria as average menstrual blood loss per period (collected over two menstrual cycles at screening and objectively measured in a laboratory) in excess of 50 mL.

The distribution of menstrual blood loss in the general population of women of reproductive age is heavily skewed with most women's MBL measuring below 50 mL per period while some experience very high levels of MBL. Informed by data from previous studies on MBL levels in the general population^13,14^ and a more selected clinical study population,^15^ we approximated this distribution using a log-normal distribution and modelled MBL levels in our simulation study on this. To be included in our simulated trials, potential participants had to have an average MBL of at least 50 mL over the two screening measurements. Measurements taken from the same subject were correlated and were modelled in a two-stage procedure:

MBL measurements from subject *i* at time point *t* were modelled as the sum of two random variables

$$Y_{\mathrm{it}}=S_{i}+E_{\mathrm{it}}$$

*S_i_* represented the underlying mean MBL level for subject *i* and was generated from the log-normal distribution referred to above. The second term, *E_it_, ∼ N(*0*, σ_e_*^2^*)* denoted the random deviation in the observed MBL at time *t* from the subject's underlying mean. We had evidence^11^ to support the assumption that *E* was approximately normally distributed with *σ_e_ = *17.9 *mL*. *S* and *E* were assumed independent at first. For each subject, we therefore simulated just one value for *S* but a separate value for *E* for every new observation thereby inducing compound symmetry in the covariance structure across repeated measurements. This approach had the added advantage that regression to the mean was naturally imposed on the simulated data as part of the enrolment process.

After collecting two simulated measurements at screening, *Y*_1_ and *Y*_2_, eligible participants (i.e. those with *(Y*_1_* + Y*_2_*)/*2* ≥ *50 mL) were enrolled in the trial and allocated at random to receive either Placebo with probability *π_p_* or one of *J − *1 active doses with equal probability *(*1 − *π_p_)/(J − * 1*)*.

Next, we added a treatment effect, *D_j__(i)_*, to participants' underlying mean MBL level according to the specified dose-response curve under the particular scenario. Having added a treatment effect we then simulated two further MBL measurements at follow up as *Y_it_ = S_i_ + D_j__(i)_ + E_it_, for t = {*3*,*4*}*.

The process of screening and randomising eligible participants, adding treatment effects and sampling MBL outcomes at follow-up was then repeated until the first interim analysis (when a certain number of participants had been randomised in accordance with the adaptation schedule). At this point we used a SAS macro^16^ to manipulate variables containing participants' randomised dose levels and MBL data from screening and follow-up into a format appropriate for parsing in WinBUGS. The interim analysis was then executed with the relevant data files and model script through a batch call in SAS.

An estimate of the dose-response curve was then computed in WinBUGS by fitting the NDLM (section 3) to the simulated interim data. Estimates of the treatment effects at each dose level were derived as the contrast *γ_j_ = θ_j_ − θ*_1_*, for j = {*2*, … , J}.*

Model parameter estimates were based on 10,000 simulated draws from the marginal posterior distributions having discarded the first 5000 iterations from the sampler (the burn-in). Convergence of the MCMC sampler was monitored for selected design-scenario combinations by sampling from two chains simultaneously using over-dispersed initial values and calculating the BGR diagnostic,^17^ coupled with visual inspection of the iteration histories.

Posterior estimates of the treatment effects, and if appropriate, other relevant quantities necessary for evaluating the utility function were then saved to text files and imported into SAS for processing. Finally, allocation probabilities were updated according to the pre-specified adaptation rules and applied to randomisations of new participants enrolling into the next stage of the trial. The process was repeated until the next interim analysis, and so on, until all 100 participants were randomised. At the end of the trial the final model estimates were derived. A successful trial was defined as one where the posterior probability that *γ_j_ > *0 was in excess of 97.5% for at least one of the active doses, *j ∈ {*2*, … , J}*. The trial outcome (success or failure) was recorded along with other parameters used for monitoring the simulations. These included the intermediate and final model parameter estimates, the number of participants randomised to each dose level overall and at each phase of the trial, and the utility function evaluated at each dose level and phase of the trial. The process was repeated 200 times for each trial design under the assumed scenario. The magnitude of the mean treatment effect estimates at each dose level was monitored to check for bias.

**Figure 3.**
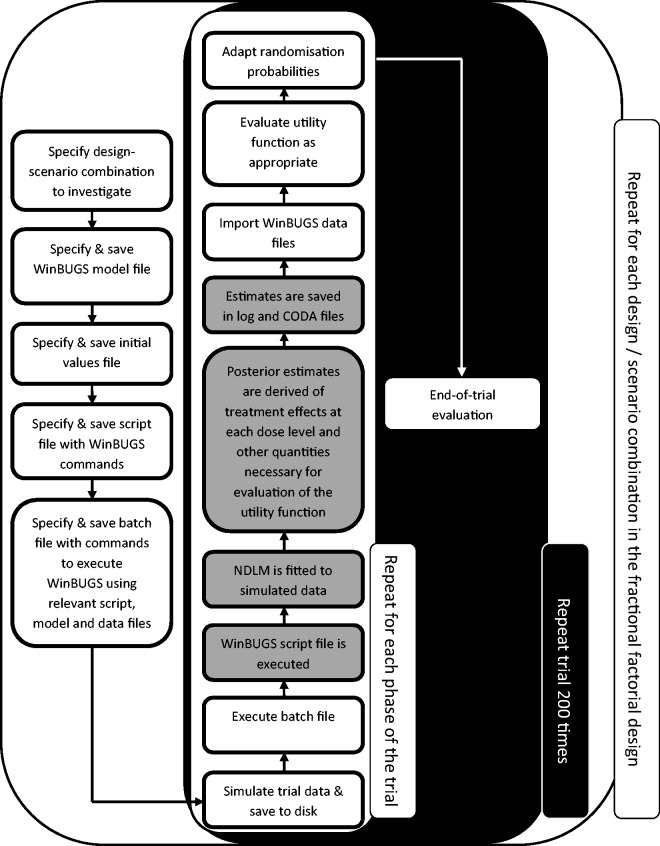
The Simulation engine. White (grey) boxes indicate tasks performed in SAS (WinBUGS).

A fully factorial design would have entailed investigating nearly 10,000 unique design-scenario combinations requiring almost 2,000,000 trial simulations. With each trial simulation taking up to 3.5 min to complete on a standard specification desktop PC, this number of simulations was not feasible. Instead, we assessed a selected 142 relevant combinations of trial designs and scenarios amounting to 1.5% of a fully factorial design. The tasks and dialogue between the SAS Software and WinBUGS are summarised in Figure 3. A detailed description of the simulation study including plans for the design options and scenarios to be assessed was reviewed by the independent data monitoring committee before the simulation was executed.

## 6 Methods for the analysis of simulation results

The changing pattern of randomised treatment allocations over the course of simulated trials was summarised graphically to illustrate the extent to which the utility function being applied at each adaptation achieved the desired modifications to the randomisation probabilities.

The quantitative design properties of interest were:

1. *Family-wise type I error rate* Under the trial scenario where no Dexamethasone dose has any benefit over placebo (a “flat” dose-response curve), the probability that the trial wrongly concludes that at least one Dexamethasone dose is efficacious.
2. *Trial power* Here we use the statistical definition of disjunctive power:^18^ the probability that, in the presence of a genuine Dexamethasone effect, the trial correctly identifies at least one Dexamethasone dose as being efficacious.

We used a normal linear model to analyse the results from the fractional factorial simulation study. We modelled the overall effect of each design option across a range of trial scenarios. Separate models were fitted for trial power (for simulated dose-response curves that included a true benefit of Dexamethasone) and type I error rate (for scenarios in which a “flat” dose-response curve was simulated). Important two-way interactions between design options and trial scenarios were investigated within the normal linear model, for example to explore whether the impact on trial power of increasing the number of adaptations differed across a range of dose-response curve shapes.

For each design option and trial scenario the mean change in statistical power relative to the reference design option or trial scenario was estimated from the normal linear model alongside its corresponding 95% confidence interval.

## 7 Results from the simulation study

**Figure 4.**
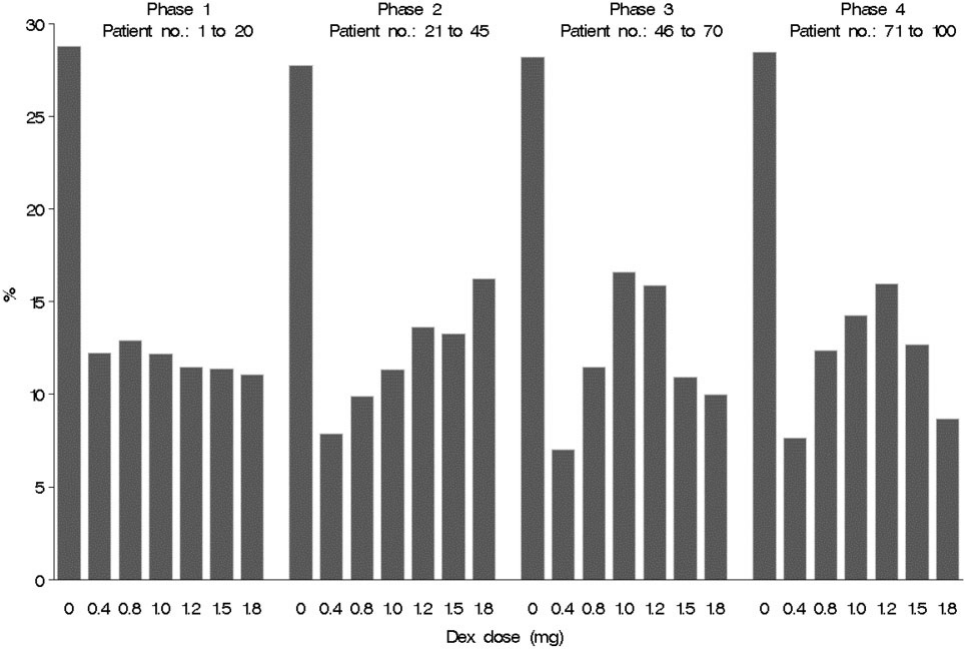
Proportion of patients randomised to each of seven trial arms during the four phases of an adaptive trial with three adaptations and fixed 28.6% allocation probability on placebo. Phase 1: before adaptation commences, equal allocation probability across all active doses. Phase 2: after adaptation #1 based on MBL outcome data collected on the first 20 patients. Phase 3: after adaptation #2 based on MBL outcome data collected on the first 45 patients. Phase 4: after adaptation #3 based on MBL outcome data collected on the first 70 patients. The data presented are the average proportions observed from 200 simulated trial runs. The most effective dose was between 1.0 and 1.2 mg.

Figure 4 illustrates the evolving pattern of randomisations for one of the types of adaptation evaluated: three in-trial adaptations after 20, 45 and 70 patients randomised. The randomisation probabilities were adapted during the course of the trial using the ‘play-the-winner’ criterion described in section 4.

**Figure 5.**
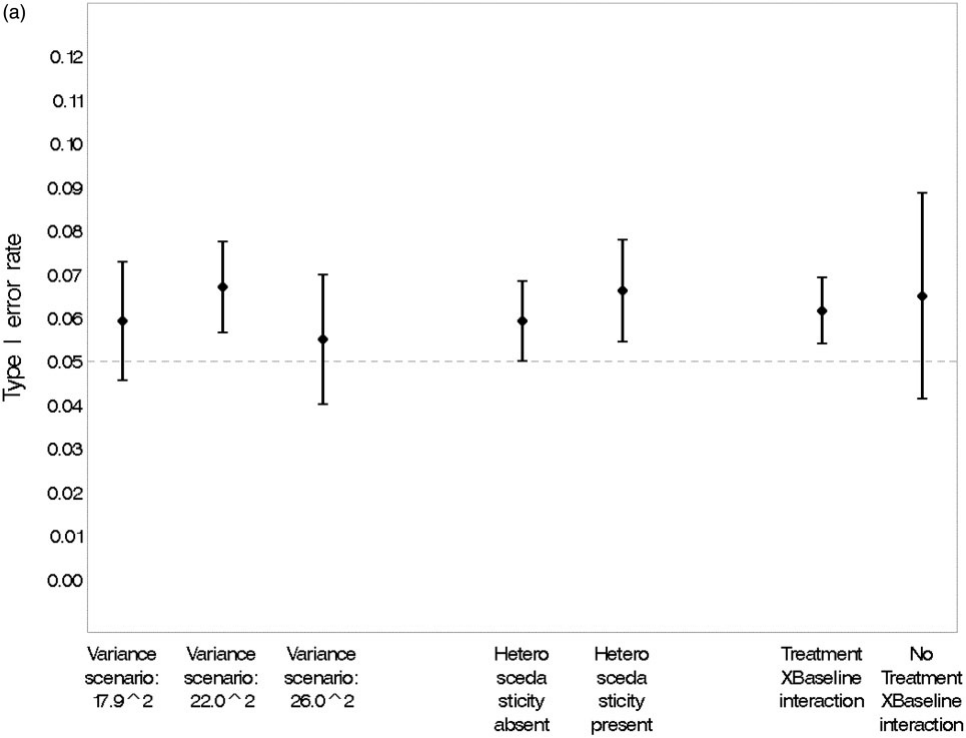

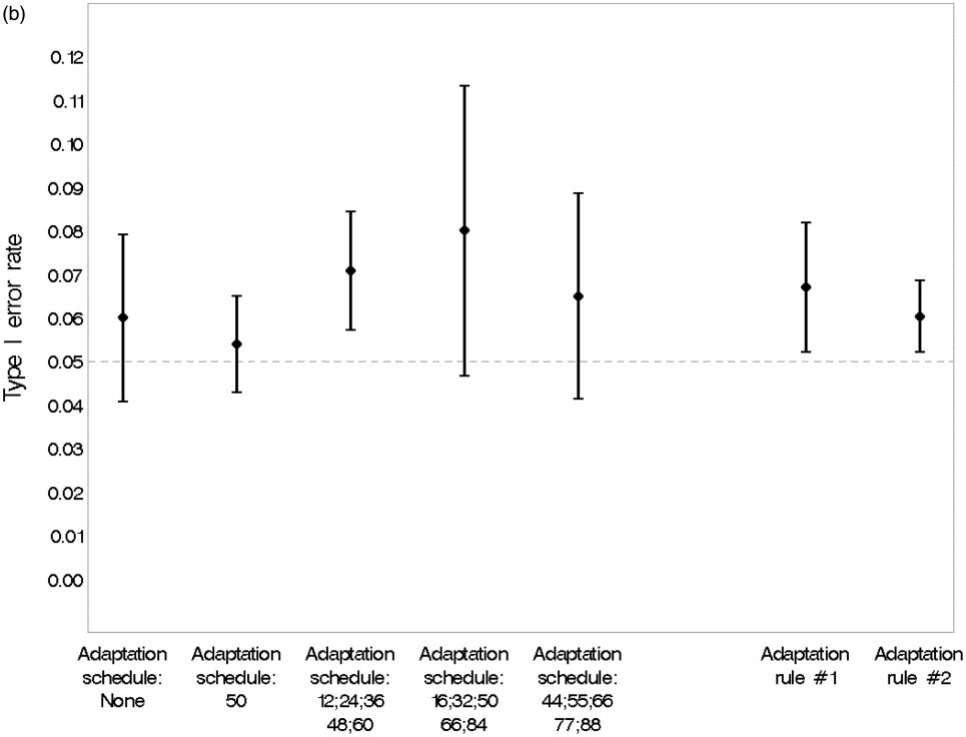

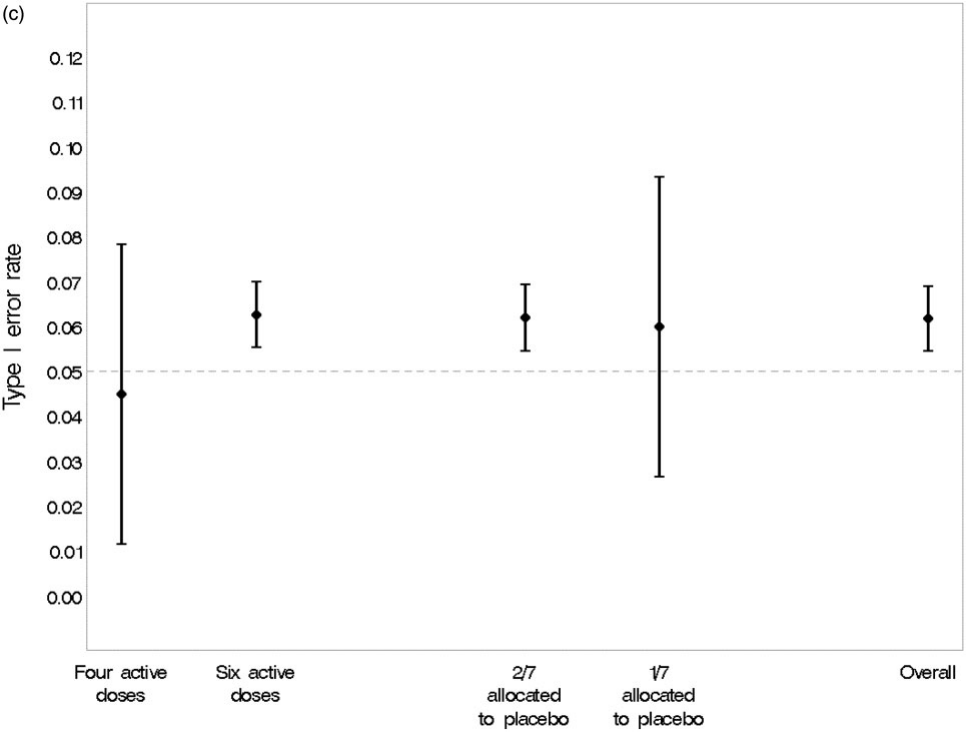
(a) Estimated type I error rate and 95% CI under various scenarios. (b) Estimated type I error rate and 95% CI under various design options. Adaptation rule #1: adapts percentage randomised to each dose in proportion to the current estimate of the probability that it is efficacious in MBL reduction. Adaptation rule #2: alters randomisation according to the precision of the estimated response at the ED95 (the minimum dose with near-maximal efficacy). (c) Estimated type I error rate and 95% CI, overall and under various design options.

Figure 5 confirms that none of the design options simulated was associated with a significant change in the type I error rate estimated from the simulations. The overall type I error rate of 6.2% (95% CI, 5.5% to 6.9%) was acceptable.

**Figure 6.**
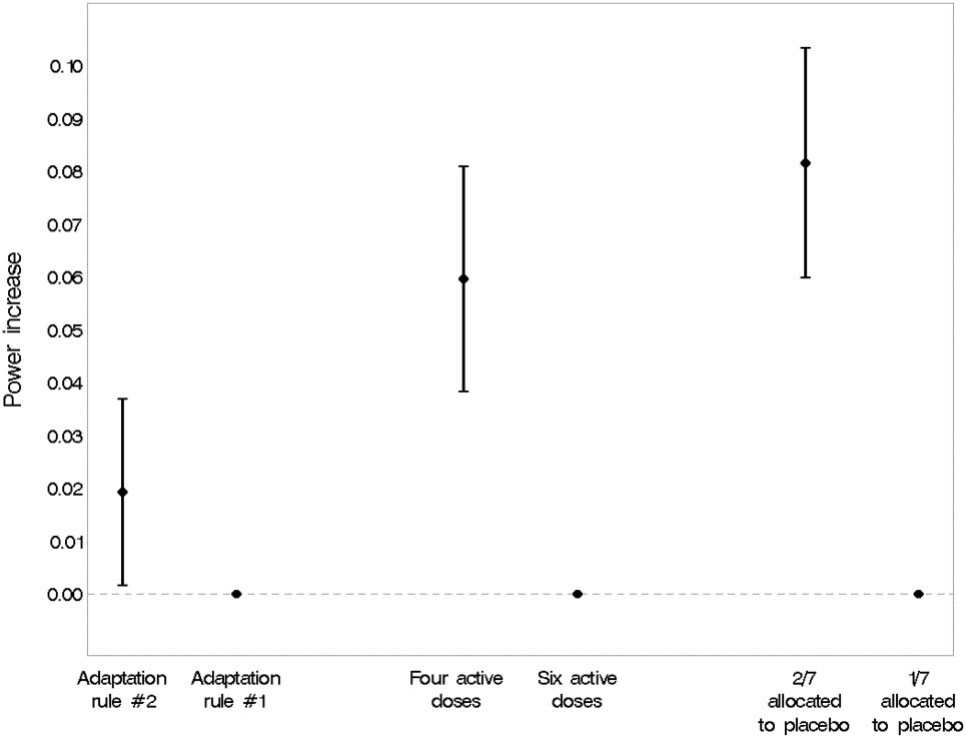
Main effects on statistical power of three design options. Estimates are averaged over all scenarios with a ‘genuine’ treatment effect. Adaptation rule #1: allocate in proportion to current probability that the treatment dose affects at least some reduction in MBL. Adaptation rule #2: based on the precision (the reciprocal of the variance) of the estimated response at the ED95 (the minimum dose with near-maximal efficacy) after one further patient has been randomised (one-step-ahead approach). The vertical bars indicate 95% confidence intervals.

Figure 6 illustrates the association between three design options and statistical power. Adaptation according to the precision of the estimated response at the ED95 (the minimum dose with near-maximal efficacy) showed a slight advantage over adaptation of allocation probabilities in proportion to the current estimate of the probability that a dose is efficacious in MBL reduction. Statistical power was greater if only four rather than six active doses were studied, although the six dose designs performed with acceptable power across scenarios. Having a higher proportion of participants randomised to placebo throughout the trial (2/7 versus 1/7) led to a substantial gain in power.

**Figure 7.**
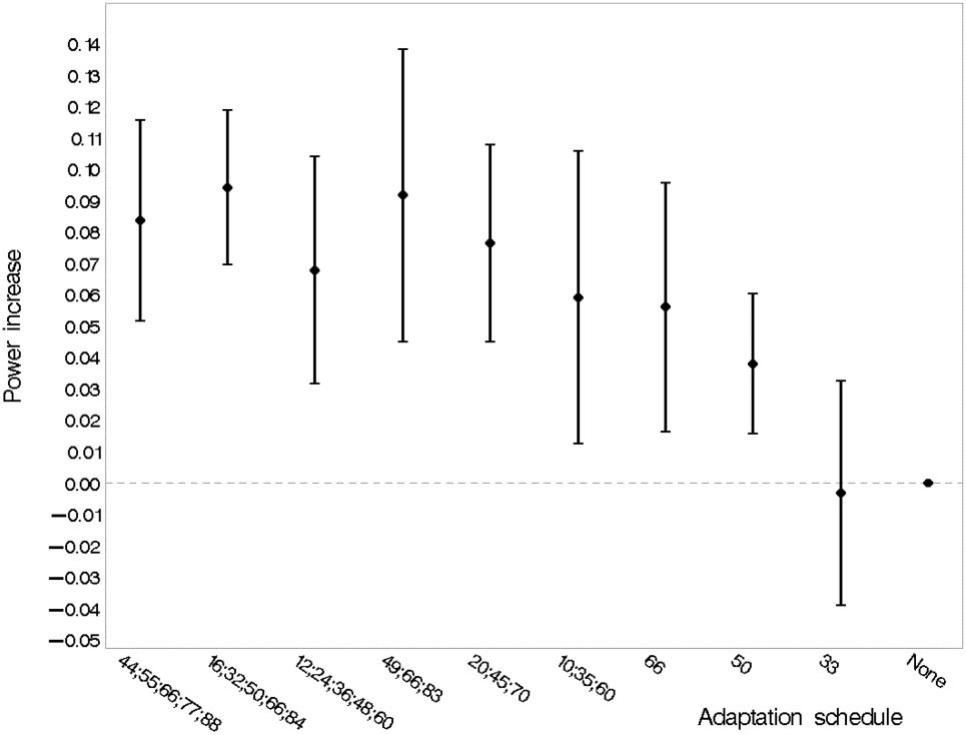
Main effect of the adaptation schedule. Estimates are averaged over all scenarios with a ‘genuine’ treatment effect. The labels along the horizontal axis indicate the number and timing of adaptations (e.g. ‘10;35;60’ is a design with adaptations after 10, 35 and 60 randomisations). The vertical bars indicate 95% confidence intervals. The reference category is ‘no adaptation’.

Figure 7 describes the performance of various adaptation schedules in terms of statistical power. Increasing the number of adaptations was beneficial; with five adaptations, spreading those evenly throughout recruitment after 16, 32, 50, 66 and 84 randomisations gave the highest point estimate of statistical power.

Table 2 reports the relative impact of various design options and trial scenarios on statistical power by listing the main effects from the normal linear modelling. Recruitment rate and presence of heteroscedasticity were not significantly associated with trial power, although some variation in power was observed for different shapes of the dose-response curve. As expected, the magnitude of treatment effect and variability in the primary outcome were the dominant influences on power. Increasing the proportion randomised to placebo, reducing the number of Dexamethasone doses studied and various schedules of adaptive randomisation also provided a benefit in terms of statistical power. No important two-way interactions were identified in the normal linear modelling.

**Table 2.** Summary of main effects of design features from normal linear modelling of simulation outputs for all scenarios containing a genuine dexamethasone effect.

		Levels	Mean power change versus reference	*P-value* ^a^
Scenario	Curve	(1) Sine curve: steep	−9%	<0.0001
		(2) Sine curve: slow	−5%	<0.0001
		(3) Non-monotone	Reference	–
	Variance	(17.9 mL)^2^	+17%	<0.0001
		(22 mL)^2^	+8%	<0.0001
		(26 mL)^2^	Reference	–
	Randomisation rate	2 pt/month	-0.7%	0.24
		4 pt/month	Reference	–
	Maximum mean effect of dexamethasone	16.4 mL	+44%	<0.0001
		8.2 mL	Reference	–
	Mechanism	No interaction	+3%	0.007
		Treatment-BL interaction	Reference	–
	Heteroscedasticity	Absent	+0.4%	0.76
		Present	Reference	–
Design option	Timing of adaptations (in terms of number of patients randomised)	33	0%	0.86
		50	+4%	0.0010
		66	+6%	0.0058
		10; 35; 60	+6%	0.013
		20; 45; 70	+8%	<0.0001
		49; 66; 83	+9%	0.0001
		12; 24; 36; 48; 60	+7%	0.0003
		16; 32; 50; 66; 84	+9%	<0.0001
		44; 55; 66; 77; 88	+8%	<0.0001
		No adaptation	Reference	–
	Utility function for adaptations	Predicted increase in precision of response at ED95 after one future randomisation	+2%	0.033
		Proportional to current probability that dose reduces MBL	Reference	–
	Doses	Four active doses	+6%	<0.0001
		Six active doses	Reference	–
	Placebo allocation rate	2/7	+8%	<0.0001
		1/7	Reference	–

a Each *p*-value is from the normal linear modelling of trial power and relates to the t-statistic comparing a given level to the reference category.

## 8 Rationale for the adaptive design selected for DexFEM

We chose the following adaptive design for the DexFEM randomised, double-blind, placebo controlled trial to identify the minimum Dexamethasone dose with near-maximal efficacy.

- five adaptations;
- adaptations spaced evenly across the randomisation period, adapting after 16, 32, 50, 66, 84 patients have been randomised;
- use of a utility function to guide the adaptations based on the precision (the reciprocal of the variance) of the estimated response at the ED95 (the minimum dose with near-maximal efficacy) after one further patient has been randomised;
- 6 dexamethasone doses (0.4, 0.8, 1.0, 1.2, 1.5, 1.8 mg total daily dose);
- 28.6% (2/7) of patients allocated to placebo throughout.

The simulation findings and other key considerations informed the design choice. Successive increases in the number of adaptations led to corresponding increases in statistical power. While there was no marked advantage of five adaptations over three, a 5-adaptation design with adaptations evenly spaced throughout recruitment was considered feasible. As well as giving fractionally the highest statistical power in simulations, the design is straightforward to communicate to collaborators. We selected the utility function providing slightly greater statistical power. The improved power of a 4-dose design over a 6-dose design is somewhat counter-intuitive. This may be because in our simulated example, the subset of four doses by chance included points on the dose response curve with greatest efficacy. We nevertheless selected the 6-dose design, because we aimed to study the largest possible number of doses while retaining acceptable statistical power. Finally, we opted to randomise 2/7 of patients to placebo as this provided much greater power than the alternative.

The statistical power of such a design estimated from the normal linear model is 93.8% (95% confidence interval 91.9% to 95.8%) for simulations on all shapes of dose response curve with a mean benefit over placebo of 16.4 mL and a within patient MBL standard deviation of 17.9 mL, averaged across heteroscedasticity (present/absent) and interaction with baseline MBL (present/absent), for a trial randomising four patients per month. For comparison, a conventional non-adaptive design studying all six doses under similar scenarios would have 82.5% power.

## 9 Discussion

### 9.1 Summary of results

We identified a Bayesian adaptive dose-finding design which had high statistical power compared with a standard parallel group design and which performed consistently regardless of trial recruitment rate, shape of dose-response curve and deviations from the model assumptions. A notable finding was the substantial gain in power from allocating a greater proportion of participants to placebo: this is consistent with what would be expected from optimal design theory and with what has been found in other parallel group designs evaluating multiple treatments.^19,6^

Our simulation studies confirmed that the use of an adaptive design conferred efficiency gains over a conventional parallel group design. The statistical power of our chosen adaptive design is substantially greater than that of a design which did not incorporate adaptations (93.6% versus 82.5%). This increase in efficiency is consistent with that found in the context of adaptive seamless phase 2/3 designs^20^ where a similar level of improvement in statistical power equated to a 25–40% saving in sample size.

The modelling of dose-response using the NDLM, coupled with the efficiency gains of our adaptive design enables a greater number of doses to be studied; otherwise one is required to select a subset of all available doses without prior knowledge of the dose response.^21^ This necessary pre-selection of doses may lead to further loss of statistical power if, by chance, the subset of doses selected does not include any of the more efficacious doses.

### 9.2 Influences on design choice

The elicitation of expert clinician opinion described in section 4 indicated that from a mechanistic perspective a monotone increasing dose response curve for Dexamethasone could not be guaranteed. This required us to consider more sophisticated modelling of dose response, rather than simply evaluating the highest feasible dose in a two-arm comparison versus placebo.

The upper limit of six on the number of doses being evaluated was dictated by practical considerations: it enables the doses under study to be reasonably closely spaced, while simplifying the drug packaging process and minimising wastage of drug for doses whose allocation probabilities are down-weighted following an adaptation.

Our simulations evaluated only two of the possible utility functions. We selected these on the basis of ease of interpretation (the “play the winner” approach) and efficiency of adaptation demonstrated in previous studies (quantifying the gain in information about the response at the current estimate of the ED95).^21,22^

Although the fractional factorial simulation study design led to confounding of main effects with higher-order interaction terms, this was not a substantial constraint as we were still able to explore two-way interactions between design options.

Given the volumetric nature of the MBL outcome, we might have expected there to be some deviations from the normality assumptions in the NDLM. However, these did not prove problematic, perhaps in part because our model studied the change from baseline and also adjusted for the baseline measurement. Had we identified evidence of non-normal errors, an alternative option would have been to model the log-transformed MBL instead.

### 9.3 Practical aspects

Although the importance sampling approach assisted with the computational efficiency of the simulations, nevertheless a period of 10 months was required to develop and conduct the simulation study. Much of this time involved preparing the suite of statistical programs and collaborating with experts in the therapeutic area to ensure that a credible set of design options and assumptions were considered in the simulation study; the actual simulations required approximately two months of processing time on a desktop PC. In part this was because a “belt and braces” approach to the importance sampling was taken, with m = 100 observations being simulated for each dose at each adaptation and 10,000 MCMC runs being performed on each. This is consistent with other work^9^ which suggested that at the stage when only a low number of patients have been randomised to a trial, larger numbers of simulated observations and MCMC runs are preferable. In a larger trial than DexFEM, or in the later stages of DexFEM itself, a smaller value of m and fewer MCMC runs should also be adequate.^9^

We implemented our simulations and adaptive design without the development of bespoke software packages, as use of SAS and WinBUGS would maximise the generalisability of our findings and the reuse of our programming code in other applications. The overall scope of our simulations (200 trials per scenario; 150 scenarios; 10,000 MCMC runs following a 5000 iteration burn-in for each model estimation step) was therefore influenced by this use of generic rather than tailor-made software which will have had an impact on computational efficiency and simulation run times. In addition to making use of readily available software, we linked programs in SAS and WinBUGS to form a single integrated package through which to deliver the entire design development study.

### 9.4 Technical considerations

The nature of the adaptive design means that not all of the placebo comparator patients are being studied contemporaneously with those randomised to a Dexamethasone dose. This is particularly notable for the doses given increased randomisation probabilities in the later stages of the adaptive design. In order to account for this, the final analysis of the trial should be stratified by adaptation stage to ensure that within each stage a contemporaneous randomised comparison is being considered.^23^ This accounts for so-called “cohort effects” that would be induced by trends in participant characteristics or protocol changes during the trial and ensures that the study retains the perspective of concurrent control.^24^ This consideration applies to a broad range of adaptive designs, including adaptive seamless designs, and is not restricted to the specific design implemented in DexFEM.

The optimal spacing of adaptations will depend on the rate at which participants are randomised to the trial^25^ as well as the length of follow up time between randomisation and assessment of outcome. Our result showing that five adaptations equally spaced throughout recruitment had marginally the greatest statistical power is consistent with the findings in adaptive seamless phase II/III designs^20^ which gave greatest adaptive seamless design efficiency when equal numbers of patients were included in phase II and phase III, rather than in the ratio 1:2 or 1:3. As our MBL primary outcome measure is available soon after treatment relative to the length of the period of recruitment to the trial, there was no requirement to seek an intermediate outcome on which to base adaptations, as recommended in situations where the primary outcome is measured following a lengthy follow-up period.^26^ An optimal group sequential testing framework is available in such a scenario.^27^

Our proposed design adapts on efficacy only, which is justifiable as Dexamethasone is already licenced and its safety profile well-established for much higher acute doses than the repeated doses for longer-term use that mimic physiological glucocorticoid secretion which are studied in DexFEM. In other situations, there would be the scope to adapt simultaneously on both efficacy and toxicity data, for example using the “trinary” ordinal outcome approach^28^ which combines these outcomes as 0: no efficacy and no toxicity, 1: efficacy and no toxicity and 2: toxicity. Thall and Cook ^29^ develop these approaches further by considering a bivariate binary outcome which incorporates both efficacy and safety information.

## 10 Conclusion

This simulation study has enabled us to develop a Bayesian response-adaptive design which maximises what can be learned about the Dexamethasone dose-response relationship from this clinical trial in heavy menstrual bleeding, substantially gaining efficiency over a standard parallel group design. The flexible approach we have reported identifies a design which performs robustly across a range of potential trial scenarios, and which remains feasible to deliver as it incorporates a manageable number of adaptations and is based on widely available statistical software. In a separate paper we will report on the technical aspects of using simulations to inform the development of an adaptive design, providing tips on how best to implement this using SAS and WinBUGS and including all of our statistical programming code.

## Appendix 1

There is evidence in Fraser et al.^11^ that MBL scores are subject to heteroscedastic error variance: women who experience greater average MBL tend to exhibit more variability in their observed MBL measurements. It was important to investigate the effects of such a mechanism and we therefore included scenarios in our work-up study which simulated separate error variances as a function of *S_i_*, the underlying mean MBL for participant *i*. Thus, individual error variances were generated as

$$\text{Var}(E_{i})=A+B(\text{exp}(S_{i}/100)-C)$$

where *E_i_* is the random deviation in the observed MBL from *S_i_*, *C* is a mean centring constant and *A* is the variance under scenarios with homoscedastic error variance (i.e. when *B* = 0). For scenarios allowing different error variances for each participant we used a value (198) for *B* which was estimated in a separate model of empirical data from the Fraser study.^11^ Heteroscedastic error variances were simulated for each of the relevant three values of A: (17.9)^2^, (22.0)^2^ and (26.0)^2^. (For completeness, the mean centring constant used was *C* = 2.837.)

For near-zero values of *S* this function for Var(*E*) yielded unrealistically small or even negative values. The function was therefore restricted to not produce values below Var(*E*) = 6. This is consistent with the variance observed amongst the patients in the Fraser study^11^ with the lowest observed mean MBL. At the other end of the scale, for values of *S* near 400 mL the variance function yielded extremely large values. Consequently the function was also restricted to not produce values above Var(*E*) = (70.7)^2 ^= 5000, which was about twice the largest observed variance in the Fraser study^11^.
